# High resolution Physio-chemical Tissue Analysis: Towards Non-invasive *In Vivo* Biopsy

**DOI:** 10.1038/srep16937

**Published:** 2016-02-04

**Authors:** Guan Xu, Zhuo-xian Meng, Jian-die Lin, Cheri X. Deng, Paul L. Carson, J. Brian Fowlkes, Chao Tao, Xiaojun Liu, Xueding Wang

**Affiliations:** 1 Department of Radiology, University of Michigan Medical School, Ann Arbor, Michigan 48109, USA; 2 Life Sciences Institute and Department of Cell and Developmental Biology, University of Michigan Medical School, Ann Arbor, Michigan 48109, USA; 3 Department of Biomedical Engineering, University of Michigan, Ann Arbor, Michigan 48109, USA; 4 Key Laboratory of Modern Acoustics, Nanjing University, Nanjing, 210093, China

## Abstract

Conventional gold standard histopathologic diagnosis requires information of both high resolution structural and chemical changes in tissue. Providing optical information at ultrasonic resolution, photoacoustic (PA) technique could provide highly sensitive and highly accurate tissue characterization noninvasively in the authentic *in vivo* environment, offering a replacement for histopathology. A two-dimensional (2D) physio-chemical spectrogram (PCS) combining micrometer to centimeter morphology and chemical composition simultaneously can be generated for each biological sample with PA measurements at multiple optical wavelengths. This spectrogram presents a unique 2D “*physio-chemical signature*” for any specific type of tissue. Comprehensive analysis of PCS, termed PA physio-chemical analysis (PAPCA), can lead to very rich diagnostic information, including the contents of all relevant molecular and chemical components along with their corresponding histological microfeatures, comparable to those accessible by conventional histology. PAPCA could contribute to the diagnosis of many diseases involving diffusive patterns such as fatty liver.

The century old practice of staining biological tissue for examination under a microscope remains the standard assessment tool for pathologists looking for signs of diseases. Unfortunately, limited by the observation depth of optical microscopy, conventional pathological procedures rely on *ex vivo* tissue processed on slides, which require time-consuming preparation procedures, often accompanied by spatial distortion generated during sample fixation and staining. Innovative imaging technologies capable of characterizing the three-dimensional (3D) micron-scale morphology and functions of unfixed and unstained tissues *in vivo* may provide an alternative to the current histopathology and lead to revolutionary new tools for biology and medicine. Besides significant advantages such as less-invasiveness, lower cost and less time-lag to diagnosis, tissue characterization *in vivo* utilizing advanced imaging technologies could also improve sampling in the body, greatly benefiting diagnosis with higher sensitivity and specificity.

The accurate pathologic diagnosis of a disease is essentially achieved by evaluating both the microscopic morphological features, namely physical microstructure, and the chemical contents of tissue^1^. Most currently available imaging technologies, however, are mono-physical and can only register either chemical components or physical microstructures in a biological tissue. Moreover, the findings from most of current imaging technologies are difficult to quantify and highly dependent on the systems and operators. Therefore, diagnostic imaging, in most cases, still cannot replace histology, nor provide similar information earlier. The noninvasive evaluation of the microstructures in deep tissue has been investigated extensively by using technologies such as quantitative-diffusion-tensor magnetic resonance imaging^2^ and ultrasound (US) spectral analysis (USSA)^3^. Validated in a wide variety of organs, USSA has successfully correlated the dimensions and distributions of the US backscatters in biological tissues to the frequency-domain power distribution of the radio-frequency (RF) US signal^4^,^5^. However, as an intrinsically mono-physics imaging modality, pulse-echo US imaging cannot interrogate the molecular components or chemical substances forming these backscattering microfeatures. Other modes of US offer further capabilities, but most offer much lower spatial resolution, as in shear wave elastography^6^. The specificity and sensitivity of the US imaging are thereby limited when identifying diseases with pathogenesis involving both microstructural and chemical changes. As a specific example, it is very difficult to differentiate liver fibrosis from steatosis based on US imaging. As both fat infiltration in hepatocytes [steatosis, as shown in the histology photograph in Fig. 1] and the collagen deposition in the extracellular spaces [fibrosis, also shown in Fig. 1] exist in non-alcoholic fatty liver diseases (NAFLD), the US backscattering in liver may be caused by either fat or collagen clusters^7^.

Conventional spectroscopic optical imaging (SOI) technologies provide approaches for evaluating the chemical components in biological tissues by measuring their intrinsic optical absorption spectra^8^,^9^. By recognizing the unique optical absorption spectrum of each chemical material, SOI over a broad spectrum could achieve quantitative chemical imaging with satisfactory sensitivity. However, as a result of the overwhelming light scattering in biological tissues, conventional SOI has only limited imaging depth. Moreover, the limited spatial resolution of SOI makes it challenging to recover histological microfeatures in subsurface tissues.

Optically (photo-) induced ultrasonic (-acoustic) imaging, namely photoacoustic (PA) imaging (PAI), is an emerging nonionizing and nonradiative imaging modality combining the advantages of both optical and US imaging^10^,^11^,^12^. In PAI, the tissue sample is illuminated by a pulsed laser. When absorbed by biological tissue, the optical energy will convert to heat and cause thermoexpansion in the tissue. The thermoexpansion leads to the vibrations of the sample which generate propagating waves at a broadband of frequencies, namely PA waves. The PA waves within the ultrasonic range can be captured by US transducers for later image reconstruction. PAI thereby enables the imaging of the optical properties in deep biological tissue at ultrasonic resolution.

Encoded in the high frequency PA signal is the spatial distribution of optical absorption properties in biological tissue. PAI thereby allows for the extension and combination of optical absorption spectroscopy and microscopic tissue architecture characterization in a single, eventually portable diagnostic system. Based on the PA measurements of each tissue specimen, a two-dimensional (2D) physio-chemical spectrogram (PCS) which integrates the power spectra of RF PA signals along the full optical spectrum can be formulated. One axis of the PCS in optical wavelengths represents the optical absorption spectrum of the tissue; the other in ultrasonic frequencies represents the spatial frequencies of the statistically repeating and optically absorbing structures in the tissue. The unique PCS of a tissue sample thereby encodes rich diagnostic information comparable to that in pathology, including the chemical components within the tissue along with their corresponding physical microstructures. Comprehensive and objective assessment of tissues, as well as diagnosis and treatment monitoring of a variety of diseases could be achieved by the analysis of PCS, namely PA physio-chemical analysis (PAPCA), without invasive and complicated biopsy procedures.

In this study, we have examined the feasibility of PAPCA in tissue characterization and its potential to realize the desired non-invasive *in vivo* biopsy through the studies on established mouse models of liver diseases. *Ex vivo* and *in situ* experiments were conducted on mice with extreme liver steatosis and fibrosis. In addition, PCS of mouse livers with progressive NAFLD, including the conditions of steatosis and fibrosis, were characterized by multivariate analysis methods to examine the feasibility of PAPCA in diagnosis and characterizing the severity of NAFLD.

## Results

The laboratory animal protocol for this work was approved by the University Committee on Use and Care of Animals of the University of Michigan. All the animal experiments in this study were carried out in accordance with the approved guidelines.

### Concept of PCS

**Figure 1:**
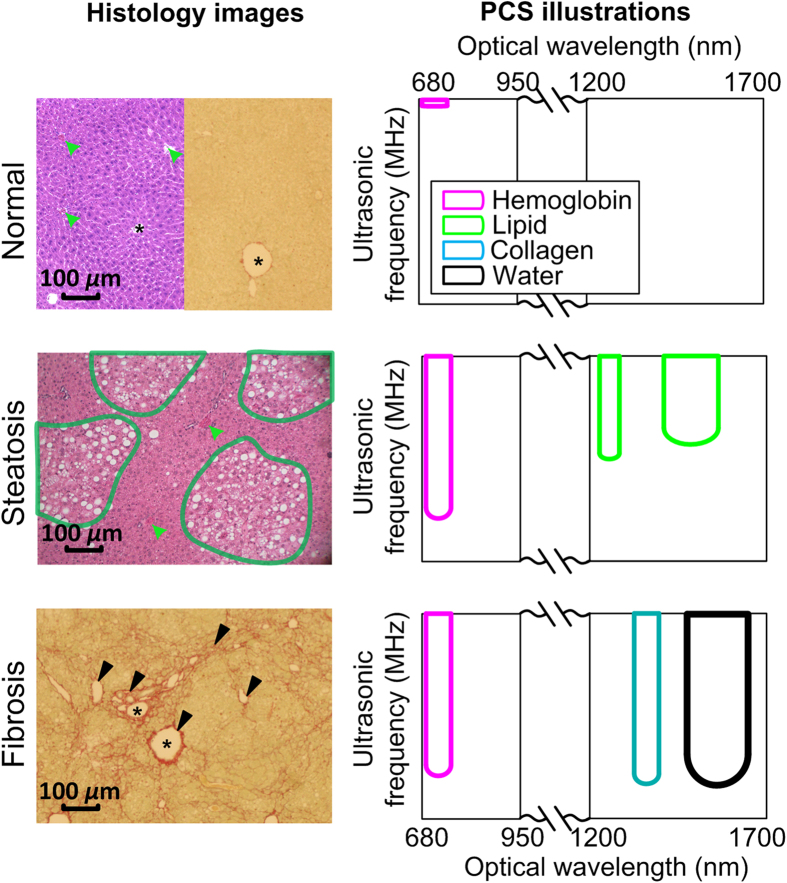
Illustration of the concept of PCS. Left column are histology photographs of normal, steatotic and fibrotic livers. Left half of the normal and steatosis histology photographs are H&E stained. The steatotic regions in the steatosis histology are outlined in green. Right half of the normal and fibrosis histology photographs are stained with Sirius Red. Red blood cells in the blood vessels and sinusoids are marked by green arrows. Fibrotic region is stained in red and marked by black arrows. In the histology photographs, “*” indicates the blood vessel or bile duct. As shown in the histology, abnormal liver conditions involve not only the changes in chemical components but also the changes in physical microstructures. Right column: the sketched PCS of the three liver conditions, including normal, steatosis and fibrosis, over the optical wavelengths covering strong absorption of hemoglobin, lipid, collagen and water. Each PCS shows distinctive signatures (i.e. combinations of the strips) which is correlated to the optically absorbing chemical components in the livers. Typically the heterogeneous distribution of a chemical component increases the extension of its corresponding fingerprint along the axis of US frequency. The concentration of a chemical component is correspondent to the magnitude of its PCS fingerprint, which will be demonstrated in Fig. 2.

Figure 1 schematically demonstrated the concept of the PCS. Similar to SOI, the optical absorption spectrum, or the “color”, of each liver specimen representing the composition of various chemical components can be observed by the PA signal amplitudes at different optical wavelengths. By putting together the power spectra of PA signals acquired at all the optical wavelengths over the entire optical-spectrum-of-interest, a PCS of a target tissue is formulated. The microstructures in the liver tissues correlate to the ultrasonic frequency domain power distribution of the PA signals, i.e., the heterogeneous distribution of the chemical components produces high ultrasonic frequency components in the PA signals. With more high ultrasonic frequency components, the power spectra appear more extended along the ultrasonic frequency axis, as shown by the vertical stripes in the 2D PCS in Fig. 1, right column. The stripe features, namely PCS fingerprints, along the ultrasonic frequency are expected at the optical absorption peaks of the major chemical components of the tissue sample. Due to the differences in chemical components and microstructures, each liver condition should possess a unique 2D PCS signature as a combination of the PCS fingerprints.

### Study on extreme models of liver steatosis and fibrosis

#### PCS of normal, extreme steatotic and extreme fibrotic livers

**Figure 2:**
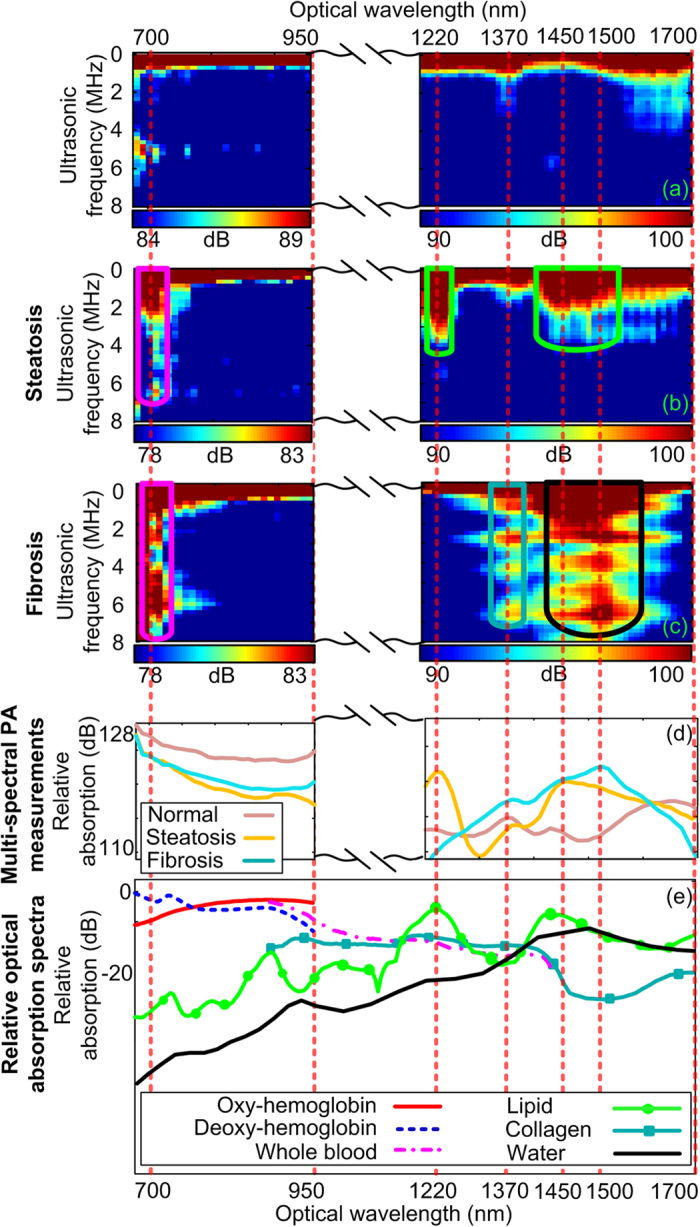
Example PCS spectrograms of normal, extreme steatosis, extreme fibrotic livers. The fingerprints of hemoglobin, lipid, collagen and water are marked by the magenta, green, blue and black contours, respectively. The dynamic range of the normal liver PCS in row 1 is maintained identical to those of the other two PCS and is adjusted to show the quick decrease of spectral magnitude along the ultrasonic frequency axis at 700 nm, i.e. less extended hemoglobin fingerprint. The multi-spectral PA measurements of normal, extreme steatosis, and extreme fibrotic livers are shown in row 4. The curves in row 4 are produced by summing the pixels in the PCS maps in normal scale along each column. The relative optical absorption spectra of the major optically absorbing chemical components in liver tissue are shown in row 5^29^,^30^,^31^,^32^. The red dashed lines indicate the matching between the absorption peaks of the chemical components and the fingerprints in the PCS.

Figure 2 shows the typical PCS of three different liver conditions, including normal control, extreme steatosis and extreme fibrosis, obtained through the *ex vivo* measurements on mouse livers. At each optical wavelength in the PCS in Fig. 2, by summing up the spectral amplitudes in normal scale from 0.1 MHz to 8 MHz [where the signal-to-noise ratio (SNR) is approximately 1], the optical absorption spectrum of each tissue sample can be obtained, as shown row 4 in Fig. 2. By looking at the unique optical spectrum of each absorbing material in row 5 in Fig. 2, one can understand the chemical contents of the three types of liver samples as combinations of hemoglobin, lipid, collagen and water.

In comparison with the normal control, the distinctive PCS of the steatotic liver demonstrates: 1) a stronger lipid fingerprint in 1200–1240 nm due to the increased lipid content in the steatotic liver (i.e. chemical change), and 2) an extended lipid fingerprint toward higher ultrasonic frequency which is a result of scattered and heterogeneous deposition of the lipid in the steatotic liver (i.e. physical microstructure change). Both findings are confirmed by the histology images in Fig. 1. Besides the changes in lipid fingerprint, the PCS of the steatotic liver also shows differences at the fingerprint associated with hemoglobin at 700 nm. Lower overall spectral magnitude (Fig. 2, row 4) and fingerprints extended into the high ultrasonic frequency (Fig. 2, row 2) are found in the PCS of the steatotic liver sample compared to that of the normal liver sample in Fig. 2. This is because the sinusoids distorted by lipid accumulation become insufficient conduits of blood, leading to the reduction of hemoglobin per microscopic field within the affected volumes in liver^13^,^14^. The distribution of such affected area within the liver volume formulated the heterogeneous distribution of hemoglobin in steatotic livers. Similar histological changes have been reported in fibrotic livers^15^.

By comparing the PCS of fibrotic liver with that of the normal control, the differences at the collagen fingerprint in 1350–1390 nm can be observed, including 1) the higher magnitude indicating the higher collagen content, and 2) further extension of the fingerprint suggesting the enhanced heterogeneity in collagen distribution in the fibrotic liver. Both of the observations are resulted from the deposition of collagen in the extracellular spaces in liver as confirmed by the histology in Fig. 1. Similar to the situation in the steatotic liver, the fibrotic liver also shows lower magnitude and further extension at the hemoglobin fingerprint around 700 nm (Fig. 2), which is due to the decrease of hemoglobin content and the heterogeneous hemoglobin distribution caused by collagen deposition.

The PCS of the steatotic and the fibrotic livers also show broad fingerprints, within the optical spectral range of 1400–1600 nm. A close observation reveals the different mechanisms causing these changes compared to the normal liver. In the PCS of the steatotic liver, the broad fingerprint peaks at 1450 nm which, according to the optical absorption spectra in row 5 in Fig. 2, is another absorption peak of lipid. Compared to the total spectral magnitude of the other two liver conditions, the fibrotic liver has larger spectral magnitude at 1370 nm and 1400–1500 nm in Fig. 2 row 4. The strong optical absorption at 1370 nm correlates to the strong optical absorption of collagen, as shown in the optical absorption spectra of the chemical components in Fig. 2 row 5. Since the fibrotic liver sample does not contain lipid deposition, the strong optical absorption within 1400–1500 nm of fibrotic is associated with the increase of water content, which possesses strong absorption in the range of 1400–1600 nm, as shown in Fig. 2 row 5. However, due to the partial overlapping of the optical absorption spectra of lipid and water, the spectral information in 1400–1600 nm will not be considered for liver tissue characterization in this study.

#### Non-invasive *in situ* imaging of mouse livers

The differences in PCS among different liver conditions raise the possibility that the lipid and the collagen fingerprints in the PCS could be the deterministic features to characterize extreme steatotic and fibrotic livers. The identification of these two liver conditions was attempted by *in situ* and non-invasive imaging experiments in mice. Since blood content is not of major concern in this experiment and the use of live animal involves strong motion artifacts, the mice were sacrificed right before the imaging experiment. The setup and procedures are described in the Online Methods section. PA signals were acquired at 1220 and 1370 nm for quantifying the lipid and collagen contents in comparison to the normal controls, respectively. The power spectra of the RF PA signals, i.e. the vertical lines in PCS at 1220 and 1370 nm, were quantified by a previously developed method, PASA^16^. The A-lines of the beamformed PA signals were segmented to formulate localized PASA parameter images.

**Figure 3:**
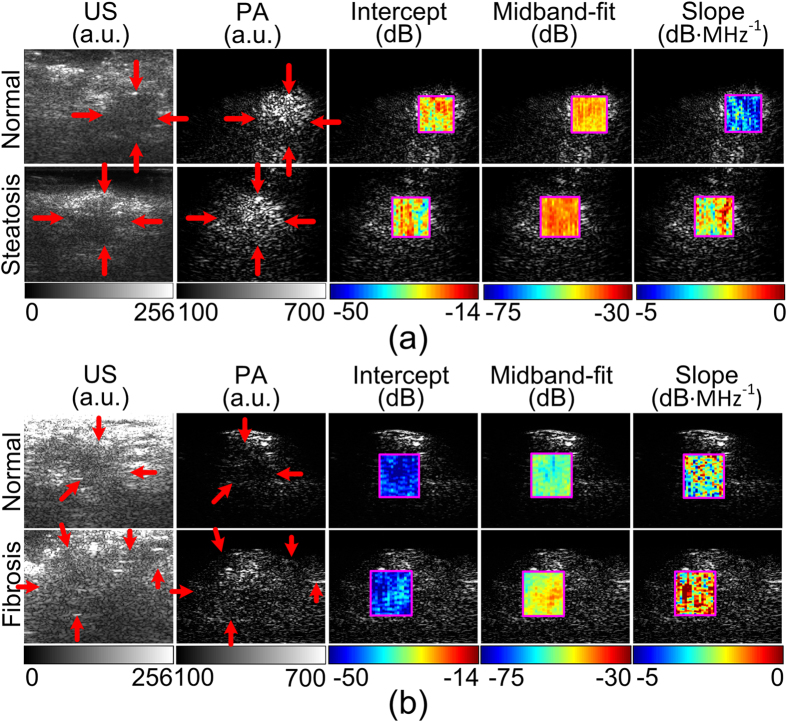
Non-invasive US and PA imaging and PASA of normal, extreme steatosis, and extreme fibrotic mouse livers *in situ*. The red arrows indicate the boundaries of the livers. The unit of the image dimensions is mm. (**a**) Normal liver vs steatotic liver at 1220 nm optical wavelength. In comparison with the normal control, the steatotic liver shows higher intercept, higher midband-fit and higher slope values. (**b**) Normal liver vs fibrotic liver at 1370 nm optical wavelength. In comparison with the normal control, the fibrotic liver shows higher intercept, higher midband fit, and higher slope values.

A commercially available linear array (L7-4, Philips Healthcare, Andover, MA) was used to cover the ultrasonic frequency range below 10 MHz, which is sufficient to distinguish the liver area from the background tissues and the hundred-micron level microfeatures in liver steatosis and fibrosis shown in histology (Fig. 1). Figure 3(a) shows the result from an obese mouse with severe liver steatosis compared to that from a normal control. In both US and PA intensity images, no obvious difference between the two liver conditions can be recognized. Comparing to the normal liver tissue, the steatotic liver shows prominently different PASA parameters, as demonstrated by each of the pseudo-color 2D PASA parameter images (intercept, midband-fit, and slope, respectively.) superimposed on the PA intensity images. The increase of lipid content in steatotic liver tissue leads to the increase of its spectral amplitude and, consequently, the PASA parameters including intercept and midband-fit; while the heterogeneous distribution of the lipid infiltrated hepatocytes increased the extension of the lipid fingerprint which is quantified as larger slope values. The contrasts in the spectral magnitudes (especially the intercept) between the normal and steatotic livers are not as prominent as those in the spectral slope. This is due to the fact that the varied thickness of the subcutaneous fat in the obese and normal mice brings in uncertainty to the attenuation of the light energy. Compared to intercept and midband-fit, the slope is less susceptible to the light fluence and, hence, is more reliable for quantitative imaging and objective tissue characterization.

The US gray-scale, PA intensity and PASA parameter images of a normal and a fibrotic mouse liver are compared in Fig. 3(b). Due to the deposition of collagen fibers and, consequently, the increase of tissue heterogeneity, the fibrotic liver shows prominent contrast in PASA parameters over the normal one at 1370 nm, including higher intercept, higher midband-fit, and higher slope.

The finding from this imaging experiment suggests that, in comparison to conventional PA intensity imaging, PASA at the optical absorption peaks of relevant chemical materials, by quantitatively and objectively assessing tissue histological microstructures, may render higher contrast when presenting different liver conditions. The extreme steatosis model, as demonstrated in this study, only involves the change of lipid content in liver; whereas the extreme fibrosis model only involves the change of collagen content. In this case, these two liver conditions can be identified by PASA of a single vertical line in PCS corresponding to the strong optical absorption wavelength of lipid or collagen, respectively.

### Study on a progressive NAFLD model

#### PCS of progressive NAFLD liver at different stages

**Figure 4:**
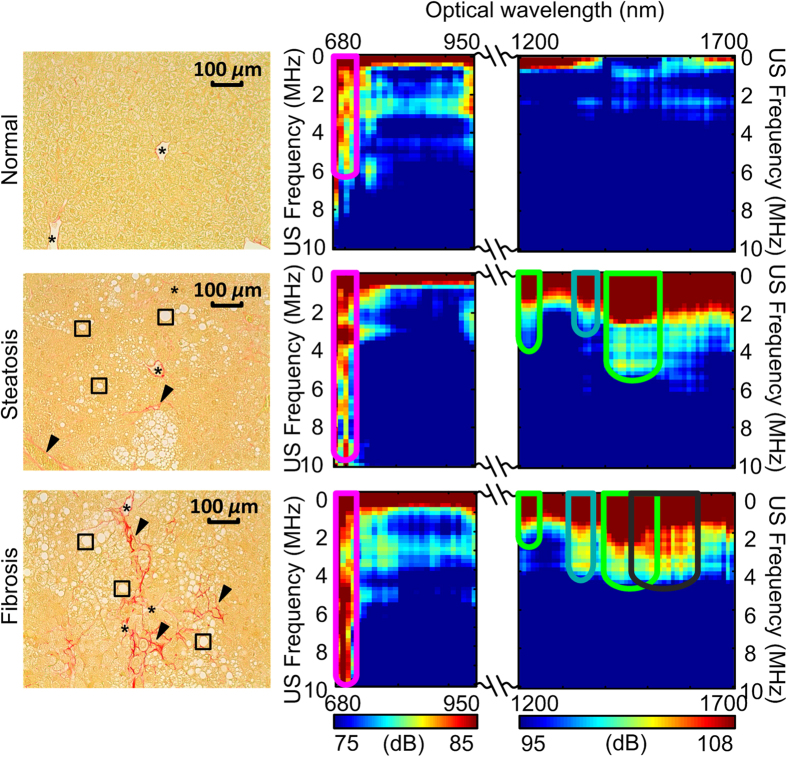
The histology photographs and PCS of the normal, steatosis and fibrosis stages in a progressive NAFLD model. In the histology photographs, “*” indicate the blood vessel or bile ducts; “◻”indicate lipid infiltrated hepatocytes; and black arrows indicate collagen content. Following Fig. 2, the fingerprints of hemoglobin, lipid, collagen and water are marked by the magenta, green, blue and black contours, respectively.

Besides the extreme cases, NAFLD in realistic scenarios involves advanced stages where steatosis and fibrosis co-exist in the liver. The non-invasive, accurate and objective grading instead of simply identifying the liver conditions has thereby greater clinical value. To further examine the sensitivity of our method to the pathological changes in liver, a mouse model (STAM, Stelic Institute and Co., Tokyo, Japan) with quick yet full spectrum of NAFLD progression was employed. The PCS of a total of 36 mouse liver specimens were acquired, including 12 normal, 12 steatotic and 12 fibrotic liver specimens. As shown by Fig. 4, the representative PCS of the three liver conditions in this progressive NAFLD mouse model demonstrate findings similar to those from the extreme steatotic and fibrotic mouse livers, including:

- (1) Both PCS of the fibrotic and steatotic livers show extended hemoglobin fingerprints at 700 nm;
- (2) Steatotic livers demonstrate enhanced and extended lipid fingerprints at 1220 nm and 1450 nm. The fibrotic livers also show discernible lipid fingerprints, corresponding to the remaining lipid contents shown in the histology images.
- (3) Fibrotic livers demonstrate enhanced and extended collagen fingerprints at 1370 nm. The steatotic livers also show noticeable yet less distinctive collagen fingerprints, generated by the small amount of collagen content shown in the histology photograph in Fig. 4.

#### Categorization of the liver conditions by Support Vector Machine (SVM)

**Figure 5:**
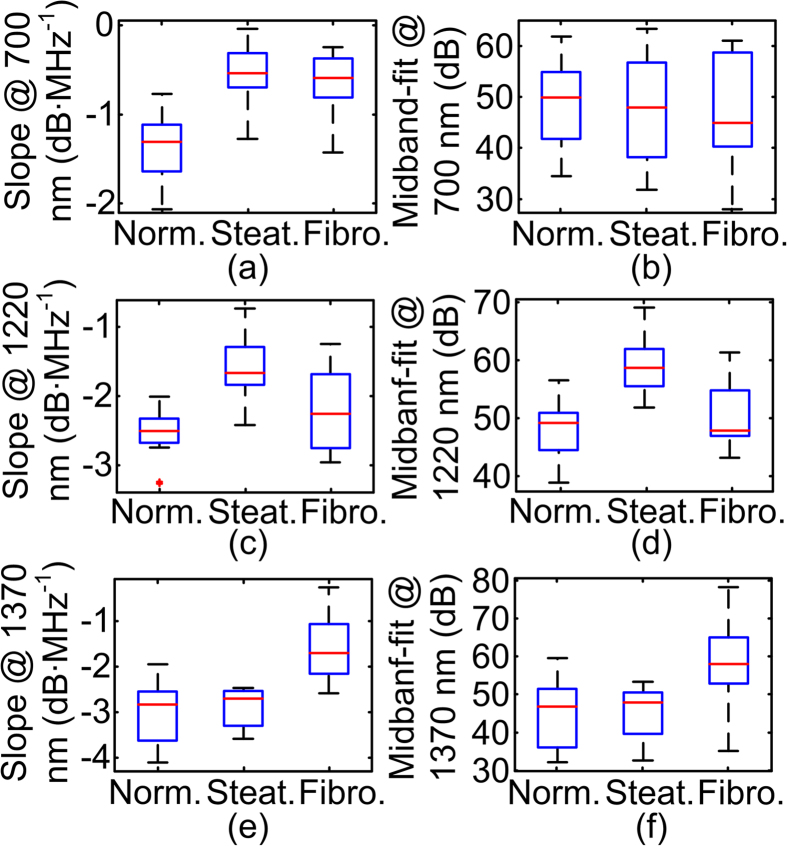
Statistics of the experiment data from the progressive NAFLD mice. Each data group consists of 12 data points. The red lines represent the averages of the data points. The upper and lower edges of the boxes are the 25th and 75th percentiles, respectively. The dashed lines extend to the most extreme data points and do not consider outliers. The outliers are plotted as “+”.

Unlike the situation in the extreme steatotic and fibrotic liver models where the histological changes involve either lipid or collagen, as shown in Fig. 1, the concurrence of steatosis and fibrosis in the different stages of the progressive NAFLD model makes these stages difficult to be distinguished. Moreover, the animal-to-animal difference further increases the challenge to establish unanimous criteria for staging NAFLD by PASA parameter at a single wavelength. Figure 5 shows the statistics of the experiment data from the progressive NAFLD mice, where the slope and the midband-fit at 700 nm, 1220 nm and 1370 nm are used independently to differentiate the three liver conditions. The average values of the PASA parameters agree well with the expected trends associated with hemoglobin, lipid and collagen changes. For example, livers at steatotic stage have high lipid content and strong heterogeneity in lipid distribution; while livers at fibrotic stage have high collagen content and strong heterogeneity in collagen distribution. Although the three groups were distinguishable by 25 to 75 percentile of the data distribution, the large deviations of and the overlaps between the groups make it difficult to accurately categorize the three groups using single PASA parameters. As examples, neither the slope nor the midband-fit values at 1220 nm can differentiate the liver steatosis and fibrosis; and the midband-fit values at 1370 nm cannot separate steatosis and fibrosis samples.

**Figure 6:**
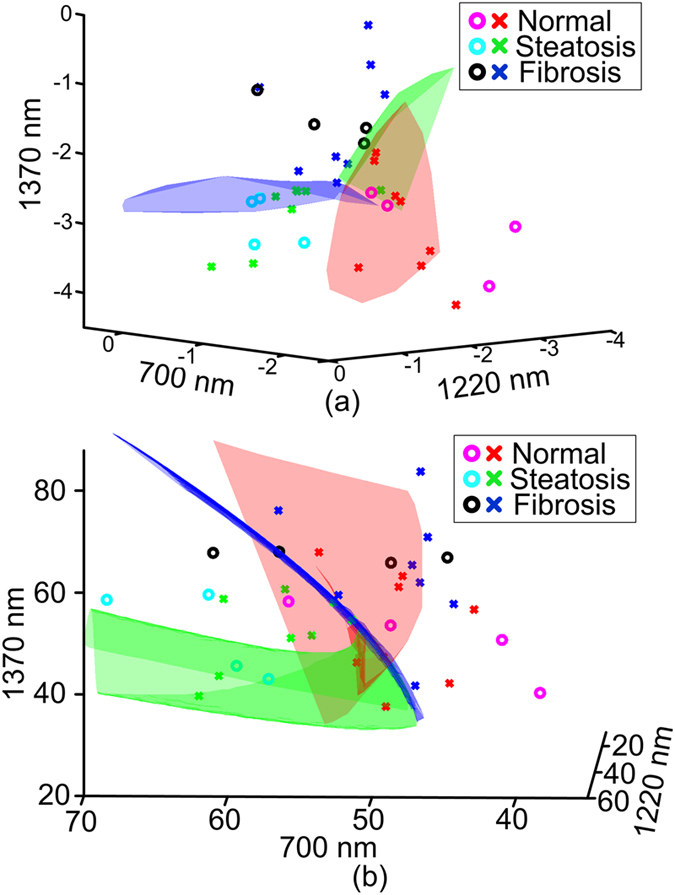
SVM categorization of the data in Fig. 5. (**a**) SVM categorization based on slopes at 700, 1220 and 1370 nm. (**b**) SVM categorization based on midband-fits at 700, 1220 and 1370 nm. “o” are training data and “x” are testing data.

PAPCA possesses the advantage of simultaneously quantifying the absolute and relative changes of a variety of chemical components in their spatial heterogeneity and concentrations. This study characterizes the physio-chemical information by implementing a multi-variant analysis tool, SVM^17^ to categorizing the PASA parameters at multiple optical wavelengths-of-interest, including 700 nm, 1220 nm and 1370 nm. A 3-fold cross-validation approach^18^ was used in this study to avoid overfitting the SVM model and to fully understand the performance of categorizing the liver conditions by SVM. The 12 sets of data acquired from each liver condition were randomly divided into 3 groups with 4 data sets. 2 of the 3 groups for each liver conditions were used in turns for training the SVM and the rest will be used for testing, that is, a total of 24 data sets (2 groups × 4 data sets × 3 conditions) for training and 12 data sets (1 group × 4 data sets × 3 conditions) for testing. As shown in Table 1, such arrangement led to three training/testing cycles for categorization with either of slope and midband-fit or both. The training and testing accuracies were averaged to evaluate the SVM categorization performance, as shown in Table 2. Figure 6(a,b) show the scattered plot of the data points and the SVM models determined in the first training cycle with slopes and midbandfits, respectively. The formulation of the SVM models is further described in the method section. As shown in Table 2, The SVM trained using only the slopes and only the midband-fits can achieve averaged accuracy of 83.3% and 50%, respectively in categorizing the test data sets. Nonetheless, when both spectral parameters are used, the accuracy can be improved to 88.9%. Such results substantiate our hypothesis that the spectral magnitude can be less reliable than the slope values due to the unpredictable nature of light energy distribution in optically-scattering biological tissues.

## Discussion

The study on the mouse models of NAFLD has demonstrated that the PCS from the PA measurement of a biological tissue contains very rich diagnostic information. Taking advantage of the endogenous contrasts of the chemical components in liver, PCS has presented not only the contents of a variety of chemical components but also the histological microstructures associated with these chemical components. Each type and condition of tissues has unique “signature” in the 2D PCS which also shows satisfying repeatability. For example, in Figs 2 and 4, steatotic or fibrotic livers induced by different pathogeneses show similar PCS. The non-invasive *in situ* experiments in Fig. 3 have also lead to the same conclusion as those in our previous study^19^. In future clinical setting, systematic analysis of the PCS, i.e. PAPCA, holds the potential for noninvasively detection and objective grading of progressive NAFLD by comprehensively evaluating both physical and chemical changes in liver tissues *in vivo*. Besides liver steatosis and fibrosis, the same method could also be extended to the diagnosis of other NAFLD related conditions such as steatohepatitis and cirrhosis. The characteristic change in steatohepatitis is the formation of macrovesicular and inflammatory foci, composed predominantly of inflammation cells (including lymphocytes)^20^. Such focal structure could be captured in PCS by implementing fluorescence agents targeted at the lymphocytes^21^. The cirrhosis induces the further loss of hemoglobin^22^, the escape of the lipid droplets^23^ and the further deposition of the collagen in liver^15^, all of which can be reflected by the change of the corresponding fingerprints in PCS. Although PAPCA was first validated through the study on liver conditions, the same technology could be adapted to the diagnosis of many other diseases including, but not limited to, kidney diseases, cardiovascular diseases or cancer where diffusive patterns of the chemical components can be found and are crucial for evaluating the severity of the diseases^5^. The diagnostic value of PAPCA could be further augmented by introducing optical contrast enhancing agents.

The liver tissues were scanned *ex vivo* and *in situ* right after the animals are sacrificed. Such approach minimizes the motion artifacts in the measurements and data averaging for optimal SNR. However, the disadvantage of using sacrificed animal is the inability to observed oxy- and deoxygenated hemoglobin individually. 700 nm illumination is thereby selected for measuring total hemoglobin optical absorption with sufficient penetration depth. In the *ex vivo* experiments, the optical and acoustic attenuation caused by the acoustic coupling materials was maintained the same for each scan by cutting the sample into same thickness. The attenuations were afterwards calibrated before the formation of the PCS following the methods described in the method section. However, for the *in situ* case, the variation of the skin and adipose thickness could bring in uncertain optical as well as ultrasonic attenuations to the measurements. Strong optical and ultrasonic attenuation could lead to low SNR, which leads to less prominent contrast in midband-fit values in Fig. 5. On the other hand, as shown in the experiment results, the slope is less affected as it describes the relative descending trend of the spectral magnitudes along the frequencies. Even though the part of the PCS could be overwhelmed by noise level, the descending trend of the spectra above the noise level could still carry microstructural information in the livers. Since the mouse skin is relatively thin, sufficient SNR has been achieved in this study. The stiffness of the livers, which could be higher in the fibrotic livers compared to the other two conditions^24^, could also bring in sample-wise variations of ultrasonic frequency dependent attenuation to the PA signals. However, since the sampling area is relatively small and the ultrasonic frequency range observed is relatively low, the stiffness of the samples was not considered and will be investigated in future studies. The optical and ultrasonic attenuation in the PA measurements will be significant in human subjects. Future studies will also include compensating these attenuations using the thickness of skin and adipose tissue in US images.

The PCS from liver tissues are characterized by performing PASA at three specific optical wavelengths corresponding to one dominant optical absorption chemical components in the liver. The PASA method facilitates quick and quantitative evaluation of both amplitudes and extensions of the PCS fingerprints, which represent the concentrations and the histological microfeatures of the relevant chemical components in the tissue samples. The PASA is performed on the linear fitting of the averaged power spectra of the RF PA signals, which provides a cogent means of addressing the stochastic nature of tissue microstructure and leads to measurements that are quantitative and repeatable. However, the linear approximation approach in the PASA does not consider the fluctuations in the signal power spectrum. Similar to the findings in USSA, the periodically fluctuating patterns in the power spectra could be another representation of the cluster sizes^25^ or repetitive distributions of the optical absorbers. The loss of varied amount of blood during the sample harvesting leads to the large uncertainty in the optical fluence within the samples and subsequently PA spectral magnitudes around 700 nm. Large variation of midband-fits in Fig. 5(b) is thereby observed. However, slopes describe the relative descending trend of PA spectra and are less affected by the blood content variation as long as sufficient SNR is achieved. The PCS fingerprints in the wavelength range of 1400–1500 nm were not included as these fingerprints contain the content and microscopic structural information of both the lipid and water. Solving for the chemical components involves decoupling both optical and ultrasonic information in the 2D PCS, which will be investigated in future studies.

The SVM used in this study is fundamentally a binary classifier, which could involve ambiguous decision^26^ when extended to multi-classification tasks. 3-fold cross-validation which uses all the data sets for training and testing in turns is used to avoid SVM overfitting and biased results. The parameter selection for optimal SVM categorization was achieved by grid search described in the user guide to the SVM package^27^. Other techniques such as feature selection may be investigated in future study for more effective SVM model optimization. SVM categorization based on neither slope nor midband-fit can achieve the accuracy when both parameters are used. This is because slope and midband-fit values are independent and provide orthogonal diagnostic information. As shown in the training/testing cycle 1 in Table 1, using only slopes, the SVM cannot correctly categorize sample 10. On the other hand, SVM trained by midband-fits successfully identified the conditions of sample 10. The slopes and midband-fits thereby compensated each other in SVM categorization using both parameters and achieved 100% accuracy. Similar observation can be found for sample 2 and 3 in cycle 2 in Table 1. The testing accuracies in most cases are lower than the training accuracies. Therefore, although 100% accuracy in cycle 1 in Table 1 has been achieved using both slopes and midband-fits, as the sample size increases, the categorization accuracy could decrease due to the individual differences and measurement noises.

For the progressive NAFLD liver model, the observation at the limited number of isolated optical wavelengths is sufficient for identifying the three stages. The relative changes among specific chemical components were not considered. Compared to conventional optical or ultrasound technologies, PAPCA possesses the unique advantage of being able to simultaneously quantifying the absolute and relative changes of a variety of chemical components in their spatial heterogeneity and concentrations which may lead to more accurate evaluation of the disease conditions. For example, the ratios between the extensions (i.e. slopes) of different fingerprints associated with various relevant chemical substances may also contain diagnostic information. Further efforts will thereby be dedicated to extracting the comprehensive diagnostic information included in the PCS with more advanced analysis tools by recognizing the entire 2D “signature” in the PCS and evaluating the contribution of each parameter to the diagnosis.

## Methods

### Mouse models

The extreme steatotic liver used for acquiring the PCS in Fig. 2 was generated in a C57BL/6J wild type mouse from Jackson laboratory. The obese mouse was fed with chow diet for the first 8 weeks, followed by 60% high-fat diet (Research diet, D12492) for the 12 weeks thereafter. The control mouse was fed with chow diet for 20 weeks. Extreme steatosis in the experiment group with no fibrosis was confirmed by histology in Fig. 1.

The extreme fibrosis model used for the PCS spectrum in Fig. 2 was FIP200 liver knock out (LKO) mouse, which spontaneously develops fibrosis without steatosis^28^. The mouse was fed with chow diet for 15 weeks. As is shown in the histology images in Fig. 1, extreme fibrosis yet no steatosis has occurred.

Both extreme models were designed to demonstrate the most distinctive features of the one liver condition in PCS without the influence of the other. For both conditions, C57BL/6J mice were used as control group.

For the categorization study, we used a mouse model which quickly develops the progressive NAFLD in liver (STAM, Stelic Institute & Co, Tokyo, Japan). As confirmed by the histological images in Fig. 4, the mouse livers formulate substantially observable steatosis at week 6 and fibrosis at week 12, although the steatosis and fibrosis level were not as high as those in Fig. 1. A total of 36 mouse liver specimens, 12 from each category of normal, steatotic and fibrotic livers, were examined in the *ex vivo* experiments.

### PCS scanning system

**Figure 7:**
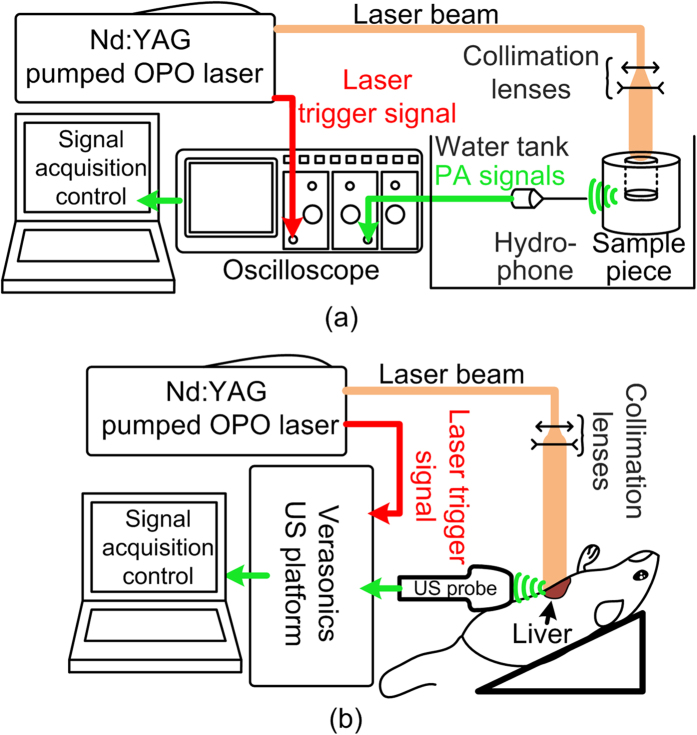
Experiment setups. (**a**) Setup for imaging of *ex vivo* liver specimens. Both the hydrophone and the sample piece were submerged in water for acoustic coupling. The laser beam was expanded by the collimation lenses to 12.5 mm in diameter and projected to the sample. The sample was held by a tube made of porcine gel (Sigma-Aldrich, St. Louis, MO). Such design minimizes the attenuation of the optical illumination by coupling water. (**b**) Setup for noninvasive imaging of mouse liver *in situ* in a model cadaver. The expanded laser beam was projected onto the mouse liver through the abdomen wall. US gel was used for acoustic coupling.

The data acquisition systems in this study are shown in Fig. 7. The optical illumination in the system was generated by a tunable Optical Parametric Oscillator (OPO) laser (Vibrant B, Opotek Inc, Carlsbad, CA, USA) pumped by the second harmonic output of an Nd:YAG pulsed laser (Brilliant B, Quantel, Bozeman, MT, USA) with spectral linewidth of 15–20 cm^−1^. The tuning ranges of the laser are 680–950 nm and 1200–1700 nm, covering the strong absorption range of hemoglobin and the vibrational absorption peaks of lipid, collagen and water in near-infrared (NIR) spectrum^29^,^30^,^31^,^32^. A constant tuning step size of 10 nm was set during the PCS acquisition. The energy output over the entire tuning range was controlled to 15–20 mJ per pulse, with a pulse duration of 8 ns. The laser beam was collimated to 12.5 mm in diameter, leading to an incident light fluence of 11–15 mJ/cm^2^ which is below the American National Standards Institute (ANSI) safety level. 10% of the laser energy was projected to an optical power meter by a beam splitter for energy monitoring and later signal magnitude calibration.

For *ex vivo* studies in Fig. 7(a), the received PA signals were collected by a needle hydrophone (HNC-1500, ONDA Co., Sunnyvale, CA, USA) with a receiving bandwidth of 0–10 MHz and sensitivity of −242 dB re 1V/*μ*Pa. The location of the sample holder as well as the hydrophone, and the receiving direction of the hydrophone are all fixed to assure minimized measurement variability. The signals received by the hydrophone was amplified and then digitized by a digital oscilloscope (TDS 540, Tektronix, Inc., Beaverton, OR, USA) before being collected by a PC. In the *in situ* experiments in Fig. 7(b), the data acquisition was conducted with our PA-US dual-modality real-time imaging system^10^. PA signals were acquired using a commercial US transducer array (L7-4, Philips, Andover, MA) covering an ultrasonic frequency band below 10 MHz. For all data acquisition, the PA signals were averaged 30 times to improve SNR.

### PCS formulation and PASA

The PA signals from the tissue generated at each wavelength, after being transformed into ultrasonic frequency domain, were calibrated by the laser energy, the frequency dependent attenuation of the acoustic signals and the system frequency-dependent response. In the *ex vivo* study, the acoustic attenuation was estimated by considering the acoustic path as the distance between the hydrophone surface and the center of the sample^16^. The quantitative attenuation was calculated using the data in a previous study^33^. The frequency response of the hydrophone was provided by the vendor. In the *in situ* study, since the US probe was attached close to the liver tissue, the acoustic attenuation cause by the skin and adipose tissue was less significant and thereby not considered. The frequency response of the US probe system was quantified by measuring the PA signals from a hair fiber^34^, which has the dimension (30 *μ*m) beyond the spatial resolution of the US probe and can be regarded as an impulse to the data acquisition system. The complete set of power spectra from all the optical wavelengths formed a PCS, as shown in Figs 2 and 4, with the optical wavelengths shown along the horizontal axis, the US frequencies shown along the vertical axis, and the intensities of the power spectra shown in pseudo-color.

For quantitative and objective analysis of PCS to achieve tissue characterization, the PA signal power spectra at the strong optical absorbing wavelengths of relevant chemical substances (e.g. hemoglobin, lipid, and collagen) were analyzed by the PASA methods as described in our previous works^16^. To be brief, the power spectrum of the PA signal from the target tissue was first fit to a linear model. Then, three spectral parameters, including intercept, midband-fit and slope, of the linear model were quantified. Among the three, only two are independent. Since both intercept and midband-fit represent the spectral magnitude, and midband-fit appears more robust to the uncertainties in measurements in Fig. 3, intercept is not included in the SVM categorization in Table 1.

### Statistical study and categorization with SVM

As mentioned in 4.1, each of the 36 mouse liver specimens was scanned, and a total of 36 PCS were generated. With each PCS, the slopes and midband-fit values at the three optical wavelengths of 700 nm, 1220 nm and 1370 nm were quantified. The mean and the standard deviation of the slopes and midband-fits within each liver condition group (i.e. normal, steatosis, and fibrosis) were calculated by the built-in functions in MATLAB (2011b, Mathworks, Boston, MA) and shown in Fig. 5. A multi-variant analysis MATLAB tool, LIBSVM^35^ was used to examine the feasibility of characterizing the liver conditions using the PASA parameters (i.e. the slope and the midband-fit at the three optical wavelengths including 700 nm, 1220 nm and 1370 nm). The PASA parameters from 8 liver specimens in each category (normal, steatosis, and fibrosis) were used to train the SVM. The PASA parameters from the rest 4 specimens in each category were used for examining the diagnosis accuracy. We used a type 1 (namely C-type in LIBSVM) SVM with 3^rd^ order homogeneous polynomial kernels^17^,^35^. The polynomial kernel is defined as:


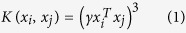


where *x* is the variable matrix, *i* and *j* are the indices of the matrix. *γ* is an adjustable parameter specific for each application. The categorization was first based on either slope or midband-fit. Afterwards, both parameters were used for improved categorization accuracy. When both parameters were used, the slope and midband-fit values were normalized within [0, 1] respectively. The capacity constant and the *γ* in the polynomial kernel were selected for each categorization case by attempting with exponentially increasing values following the user guide to LIBSVM^35^ and shown in Table 1. As previously mentioned, 3-fold cross-validation approach^18^ was used in this study to avoid overfitting the SVM model and to fully understand the performance of categorizing the liver conditions by SVM. The training and testing accuracies were averaged to demonstrate the SVM categorization accuracy, as shown in Table 2.

## Additional Information

**How to cite this article**: Xu, G. *et al.* High resolution Physio-chemical Tissue Analysis: Towards Non-invasive *In Vivo* Biopsy. *Sci. Rep.* **6**, 16937; doi: 10.1038/srep16937 (2016).

## Figures and Tables

**Table 1:** SVM categorization.

Testsamplecondition	Cate. #	Test samp.#	Cycle 1			Cycle 2			Cycle 3		
			Slopec = 3γ = 1/6	Midc = 10γ = 1/9	Bothc = 3γ = 1/2	Slopec = 5γ = 1/8	Midc = 10γ = 1/4	Bothc = 60γ = 1/2	Slopec = 10γ = 1/6	Midc = 10γ = 1/6	Bothc = 100γ = 1/5
Normal	1	1	1	1	1	1	1	1	1	3	1
	1	2	1	2	1	2	1	1	1	2	1
	1	3	1	1	1	2	1	1	1	1	1
	1	4	1	1	1	1	1	1	1	1	1
Steatosis	2	5	2	2	2	2	1	1	2	2	2
	2	6	2	2	2	2	3	2	2	1	2
	2	7	2	2	2	2	1	2	2	1	2
	2	8	2	3	2	2	3	2	1	1	1
Fibrosis	3	9	3	3	3	3	3	3	3	3	3
	3	10	1	3	3	3	1	3	3	1	3
	3	11	3	1	3	3	1	3	1	1	1
	3	12	3	2	3	3	3	3	2	1	1
Training accuracy			95.8%(23/24)	100%(22/24)	100%(24/24)	95.8%(23/24)	100%(24/24)	95.8%(23/24)	100%(24/24)	100%(24/24)	100%(24/24)
Categorization accuracy			91.7%(11/12)	66.7%(8/12)	100%(12/12)	83.3%(10/12)	50%(6/12)	91.7%(11/12)	75%(9/12)	33.3%(4/12)	75%(9/12)

A 3-fold cross-validation has been performace by using all the data sets in turns for training and testing the SVM model. The data sets are randomly devided into 3 groups. Each cycle uses 2 groups for training and 1 group for testing c is capacity constant and *γ* is the parameter in Eq. (1) for the polynomial kernel. Cate. = category. samp. = sample

**Table 2:** SVM categorization accuracy.

	Slope	Mid	all
Mean Training accuracy	97.2%	97.2%	98.6%
Mean Training accuracy	83.3%	50%	88.9%

The numbers are derived by averaging the training and testing accuracies with the same data combination for all 3 cycles in Table 1.
